# Low-Power RFED Wake-Up Receiver Design for Low-Cost Wireless Sensor Network Applications

**DOI:** 10.3390/s20226406

**Published:** 2020-11-10

**Authors:** David Galante-Sempere, Dailos Ramos-Valido, Sunil Lalchand Khemchandani, Javier del Pino

**Affiliations:** Institute for Applied Microelectronics (IUMA), University of Las Palmas de Gran Canaria (ULPGC), 35017 Las Palmas de Gran Canaria, Spain; dgalante@iuma.ulpgc.es (D.G.-S.); dramos@iuma.ulpgc.es (D.R.-V.); sunil@iuma.ulpgc.es (S.L.K.)

**Keywords:** wake-up receiver (WUR), radiofrequency envelope detector (RFED), tuned-radiofrequency (tuned-RF), low power, energy efficient, wireless sensor network (WSN), complementary metal-oxide semiconductor (CMOS), active inductor, integrated transformer

## Abstract

The development of wake-up receivers (WuR) has recently received a lot of interest from both academia and industry researchers, primarily because of their major impact on the improvement of the performance of wireless sensor networks (WSNs). In this paper, we present the development of three different radiofrequency envelope detection (RFED) based WuRs operating at the 868 MHz industrial, scientific and medical (ISM) band. These circuits can find application in densely populated WSNs, which are fundamental components of Internet-of-Things (IoT) or Internet-of-Everything (IoE) applications. The aim of this work is to provide circuits with high integrability and a low cost-per-node, so as to facilitate the implementation of sensor nodes in low-cost IoT applications. In order to demonstrate the feasibility of implementing a WuR with commercially available off-chip components, the design of an RFED WuR in a PCB mount is presented. The circuit is validated in a real scenario by testing the WuR in a system with a pattern recognizer (AS3933), an MCU (MSP430G2553 from TI), a transceiver (CC1101 from TI) and a T/R switch (ADG918). The WuR has no active components and features a sensitivity of about −50 dBm, with a total size of 22.5 × 51.8 mm^2^. To facilitate the integration of the WuR in compact systems and low-cost applications, two designs in a commercial UMC 65 nm CMOS process are also explored. Firstly, an RFED WuR with integrated transformer providing a passive voltage gain of 18 dB is demonstrated. The circuit achieves a sensitivity as low as −62 dBm and a power consumption of only 528 nW, with a total area of 634 × 391 μm^2^. Secondly, so as to reduce the area of the circuit, a design of a tuned-RF WuR with integrated current-reuse active inductor is presented. In this case, the WuR features a sensitivity of −55 dBm with a power consumption of 43.5 μW and a total area of 272 × 464 μm^2^, obtaining a significant area reduction at the expense of higher power consumption. The alternatives presented show a very low die footprint with a performance in line with most of the state-of-the-art contributions, making the topologies attractive in scenarios where high integrability and low cost-per-node are necessary.

## 1. Introduction

The development of circuits and systems for wireless communications and their applications have been under extensive research during the last decades. More precisely, the applications of wireless sensor networks (WSN) have received a lot of attention since they are one of the enabling technologies promoting the Internet-of-Things (IoT) or Internet-of-Everything (IoE) [1]. WSNs are composed of battery-powered devices operating with very low-power consumptions so as to ensure a long lifespan of the network. This requirement is crucial in many application scenarios since the batteries of the sensor nodes are replaceable at a very significant cost because of the harsh environmental conditions. The conventional radio interface or transceiver is frequently the most power-consuming element in a WSN node, dominating both the static and dynamic power consumption of the sensor [2]. For this reason, various strategies have been explored to minimize the activity of the conventional transceiver, and can result in significant energy savings, thus, increasing the sensor’s battery life. One well-known solution to drastically reduce power consumption is to aggressively duty-cycle the transceiver in order to save power, but this may leave the sensor node unreachable and unable to communicate with other nodes for a short time period [3]. Therefore, this decrease in power consumption when the sensor node’s radio is in sleep-mode results in a higher latency. Generally, two types of applications can be distinguished in terms of the network’s activity: low-average throughput applications, where the activity rate is low and power consumption and sensitivity are key metrics; and high-average throughput applications in which the network is very active and data rate is crucial. Considering the two scenarios, the delay associated with duty-cycled transceivers can severely degrade the performance of the whole WSN in the context of high-throughput applications with a high level of activity, and many real-time applications may not tolerate such delays.

**Figure 1:**
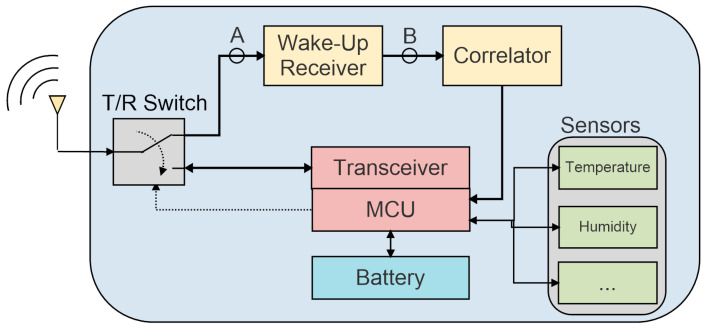
Block diagram of a sensor node integrating a wake-up receiver (WuR).

The implementation of a wake-up receiver (WuR) apart from the main radio interface can ensure a low latency along with a very low power consumption. With this solution, the WuR is always listening to the communication channel while the main transceiver remains in sleep-mode. The WuR must be constantly listening to the communication channel to be able to detect wake-up signals coming from other WSN nodes in order to maintain a reasonable latency. A duty-cycled WuR can also be used to further reduce the power consumption, at the expense of a higher latency [4]. The nodes can be individually addressed with the exchange of wake-up signals or packets, employing distinctive identifiers to avoid waking up other sensor nodes. To be able to identify the wake-up signal unambiguously and avoid the generation of false wake-ups, a WuR usually implements a digital baseband (DBB) with a pattern recognizer. One of the most frequent modulation schemes employed for WuR signals is the On/Off keying (OOK) scheme, mainly due to the simple architecture it requires for demodulation [5]. Only after reception of the wake-up signal with the right identifier are the main microcontroller (MCU) and the conventional radio interface awakened to begin data exchange via conventional radio communications. A common integration of a WuR in a WSN node is shown in Figure 1.

The most significant power consumption savings are observed when WuRs are used in low-traffic and less-dense WSNs, mainly because the main transceiver is in sleep-mode most of the time [1]. When designing a WuR, it is hard to meet high performance requirements due to the simple architecture used. Designing WuRs with a high sensitivity and data rate while keeping a low power consumption can be a challenging process.

The most noteworthy advantage introduced by the use of WuRs is the significant reduction in power consumption and low latency. Power saving is achieved by simply putting the MCU and the conventional transceiver in sleep-mode and, since the WuR remains always active, the receiver latency is not severely affected. Nevertheless, these advantages come at the cost of a higher system complexity, size and cost for the node implementation [5]. In contrast with conventional radio receivers, a WuR is a simple radio interface characterized by very low power consumption but also with a limited performance. Because of the required low power consumption, aspects such as WuR sensitivity and selectivity are often inferior to common receivers. When that is the case, a higher power is required from the transmitter to reach the WuR in comparison with the power needed to reach a conventional RF transceiver for the same distance. Nevertheless, a high sensitivity/power-consumption ratio is often unnecessary in densely populated networks since the design effort is completely focused on obtaining the lowest cost-per-node possible. Consequently, aspects such as system integrability, die footprint, bill of materials, assembly costs and calibration schemes are prioritized in such applications.

The development of WuRs has lately received much interest from both academia and industry researchers, suggesting that the integration of WuRs in commercial applications is increasing. Many state-of-the-art works have already reported designs operating well below 50 μW, and even in the sub-nW range, with very high sensitivities. This power consumption is severely lower than that of conventional transceivers, operating in the range of mW. These WuRs can be powered by coin-sized batteries and are capable of constant channel listening for several years. Some research even shows the implementation of battery-less WuRs by applying energy harvesting techniques to obtain a stable DC-supply from RF signals to feed all the building blocks [6]. Additionally, the implementation of WuRs with low-power sensors can be used to awake the transceiver and the MCU under certain environmental conditions or the triggering of some particular events [2]. This can potentially result in a more efficient performance and a reduction of power consumption if the probability of false wake-ups is kept low. Envelope detectors formed by conventional rectifiers are commonly used to implement WuRs due to the very low power consumption they allow, since no local oscillators and phase-locked loops are needed [2]. 

It can be seen in literature that a trade-off between power consumption and sensitivity exists. One of the most relevant metrics to take into account is the cost-per-node associated with each solution. Some simple indicators of cost are the technology process complexity and minimum gate length, the integrability of the design, the list of materials and assembly costs, calibration schemes if any, and die footprint. In WSNs the feasibility of integrating the WuR with complex sensors and systems is a key factor. The architecture selection plays a critical role when designing a WuR since some topologies are leaned towards higher power savings at the expense of sensitivity, and vice versa. The main distinction lies in the implementation of active RF amplifiers and mixers, achieving very high sensitivities but with a power dissipation in the order of tens of μW. Similarly, sub-nW designs are possible by avoiding RF amplifiers and mixers, but the sensitivity is then limited. 

In previous literature, different architectures provide sensitivity optimizations without a major reduction in circuit area and costs-per-node, which are of upmost importance for system integration and low-cost densely populated networks. This paper has three major contributions, since we present the development of different WuRs based on the envelope detection architecture with various modifications to improve the footprint of the circuit. In Section 2, we present the implementation of a WuR with a passive envelope detector and off-chip components in a PCB fashion. The development of two integrated WuRs in a CMOS commercial technology is shown in Section 3. Section 4 evaluates the performance of the proposed solutions and gives a comparison between the most relevant state-of-the-art solutions. Finally, conclusions of this work are drawn in Section 5.

## 2. Envelope Detector WuR with Off-Chip Components

Most works on conventional receivers are based on the Zero-IF or Low-IF direct conversion architectures [7,8,9,10]. However, the Zero-IF topology is rarely used to implement a WuR due to the presence of flicker noise and DC offsets after signal down-conversion. Hence, the Low-IF architecture is frequently preferred to overcome these two issues. Besides the conventional Zero- and Low-IF architectures, a wide range of topologies exists in the field of WuR design, such as the radiofrequency envelope detection (RFED), matched-filters (MF), superheterodyne (SH), subsampling (SS), injection-lock (IL), uncertain-IF (UIF) or the super-regenerative (SR) architectures. 

On the one hand, structures such as the SH architecture can be of interest in terms of sensitivity and interference resilience, since it is possible to implement highly selective, low-power IF filters that can reduce the impact of noise and blockers [11]. However, these WuRs suffer from very high power consumption, and an example can be found in [12], reporting a sensitivity of −97 dBm at 10 kbps with a power consumption of 99 μW. The UIF scheme has the advantage of suppressing the need of phase-locked loops (PLLs) [13], thus achieving significant power savings. This comes at the cost of requiring a very high-Q input filter to be able to resist interferers [14]. An example is reported in [15], with a sensitivity of −55 dBm at 50 kbps and a power consumption of 100 μW. Another solution that does not require PLLs is the IL architecture, employing a tuned oscillator as a frequency to amplitude converter [16,17,18]. The SR based architecture also generates oscillations depending on the input signal strength, and is characterized by an RF oscillator changing between stable and unstable states, controlled by a low-frequency quench oscillator [19]. Some examples can be found in [20,21]. The SS architecture is somewhat similar to the SH, but in this case the mixers are substituted by sample-and-hold (S/H) circuits [13]. In [22], a dual-mode receiver based on the SS principle with a sensitivity of −78.5 and −75 dBm with a power consumption of 16.4 and 22.9 μW for the two modes (10 and 200 kbps data rates, respectively) is reported. 

On the other hand, the RFED is one of the architectures with the highest potential for integrability and low-power operation of all the alternatives discussed. A substantial number of WuRs are based on this architecture [2,6,11,23,24,25,26,27,28], mainly because it allows the designer to achieve very low-power consumptions. Nevertheless, the RFED architectures are characterized by a high noise figure and low interference resilience when compared to other WuR architectures, thus, achieving a limited sensitivity. In this section, we explore the implementation of a WuR with off-chip components to demonstrate that a passive RFED WuR can achieve a reasonable performance with no DC-power consumption.

The sensor node is intended to operate in the 868 MHz industrial, scientific and medical (ISM) band with an OOK modulation scheme for the wake-up signal. A block diagram of the system is depicted in Figure 1. In this figure, node A corresponds to the OOK-modulated input signal, and at node B the OOK signal’s envelope is obtained, containing the wake-up pattern which is decoded by the next block, the pattern recognizer or signal correlator. In this architecture, there are two paths for the signal coming from the antenna. The path leading to node A is used to receive the wake-up signal whilst the main path is used for conventional radio communications. The T/R switch, configured by an external microcontroller (MCU), enables a certain path depending on the state of the sensor node. Before the low-power sleep-mode is enabled, the MCU sets the switch to activate the wake-up signal detection path. After the WuR module, the signal’s envelope arrives at the pattern recognizer, operating in the frequency range of 15 to 150 kHz. When the wake-up signal identifier matches the sensor identifier, the pattern recognizer generates an interrupt to wake up the MCU and conventional radio communications commence. The wake-up signal is generated from a low-frequency signal (0.5–4 kbps) modulated with a high-frequency carrier (15–150 kHz). The signal is then modulated with an 868 MHz carrier to be able to use the same antenna as the main radio. In this manner, a low-cost, low-power circuit, such as the AS3933 circuit operating at 125 kHz, can be used as pattern recognizer [11]. With this approach, the designer can take advantage of the propagation characteristics of high-frequency signals with the low-power capabilities of the low-frequency circuitry. 

### 2.1. Schematic Design

**Figure 2:**
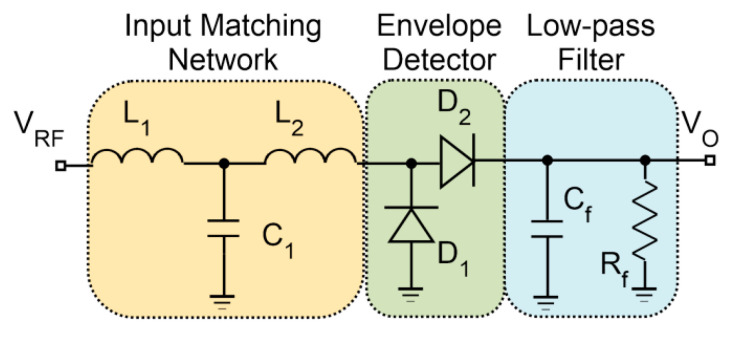
WuR architecture of the envelope detector with discrete components.

The structure of the WuR is based on the RFED principle, composed of an input matching network, a rectifier and a low-pass filter. All the components used in the implementation are commercially available off-chip components. Figure 2 shows the WuR front-end composed of a T-type LC impedance matching network, a rectifier and a conventional RC low-pass filter. 

**Figure 3:**
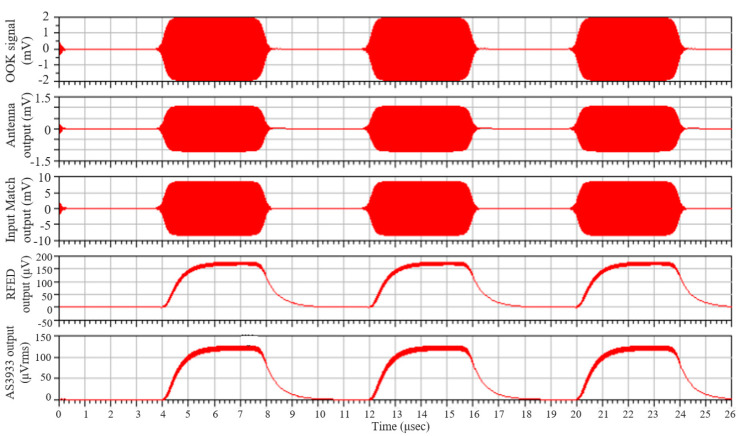
Results of the determination of the overall WuR sensitivity. A 2 mV_peak_ On/Off keying (OOK) signal is generated and passed through the antenna model and the radiofrequency envelope detection (RFED), finally reaching the AS3933 model. The plotted waves represent, from top to bottom, the generated OOK signal and the outputs obtained after the antenna, the input matching network, the RFED and the AS3933.

The rectifier is implemented with Schottky diodes due to their high-speed switching capabilities. In addition, these diodes have a very low threshold voltage, an ideal characteristic for high-frequency, low-power applications, allowing the design of a high sensitivity envelope detector. The HSMS-285X Schottky diodes from Avago were selected, since these devices are suitable for applications below 1.5 GHz and sensitivities better than −20 dBm. The design specifications are extracted from the minimum sensitivity of the AS3933 IC, which is around 80 μV_rms_ or 113.15 μV_peak_. The corresponding sensitivity of the WuR is determined to be −50 dBm from simulations, as seen in Figure 3. To achieve impedance matching at the input, L_1_ is determined to be 6 nH, L_2_ is 90 nH and C_1_, 1.2 pF. With these values, an input return loss better than 35 dB is observed in simulations at the frequency of 868 MHz. Similarly, the low-pass filter components needed, C_f_ and R_f_, have a value of 200 pF and 5.3 kΩ, respectively.

### 2.2. Implementation and Measurements

**Figure 4:**
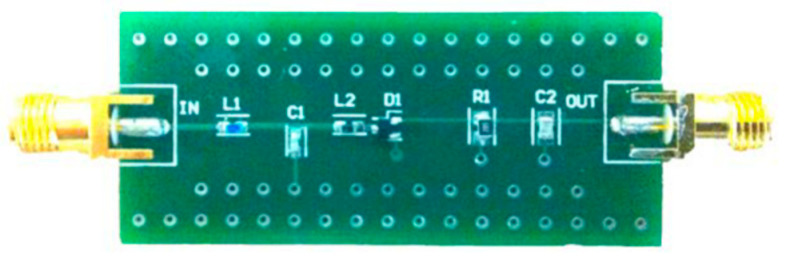
PCB implementation of the simple RFED WuR implemented with discrete components.

The RFED based WuR is implemented on a PCB to verify the simulation results. The FR-370HR substrate from Isola was selected since it offers a reasonable trade-off between cost and performance at the frequency of interest. The PCB is shown in Figure 4, with a size of 22.5 × 51.8 mm^2^.

**Figure 5:**
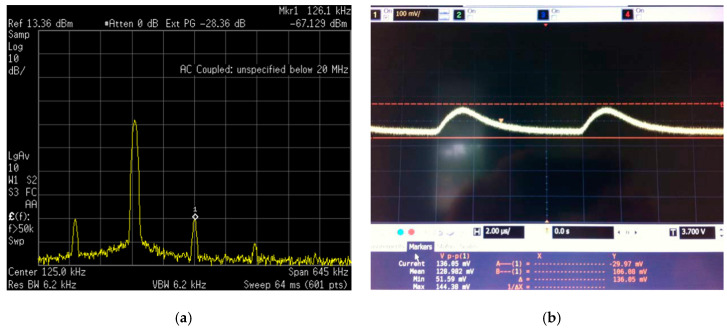
Output of the WuR in the discrete spectrum analyser for a −40 dBm AM input signal (**a**) and output of the WuR in the time domain (**b**).

An AM signal with a 100% modulation index at a carrier frequency of 868 MHz and a modulator of 125 kHz are used for measurements. The E4440A spectrum analyzer was employed to extract the results. The measured response at the output of the circuit is presented in Figure 5a, obtained with the spectrum analyzer for a −40 dBm input signal. It is seen that the output presents a 126.1 kHz signal with a magnitude of −67.1 dBm over a 50 Ω load. After removing wire losses, a value of −65.8 dBm is obtained at the RFED’s output over a 50 Ω load, which is enough to reach the AS3933′s sensitivity. The measured output of the WuR in time domain is presented in Figure 5b, representing the envelope of the 125 kHz AM signal.

**Figure 6:**
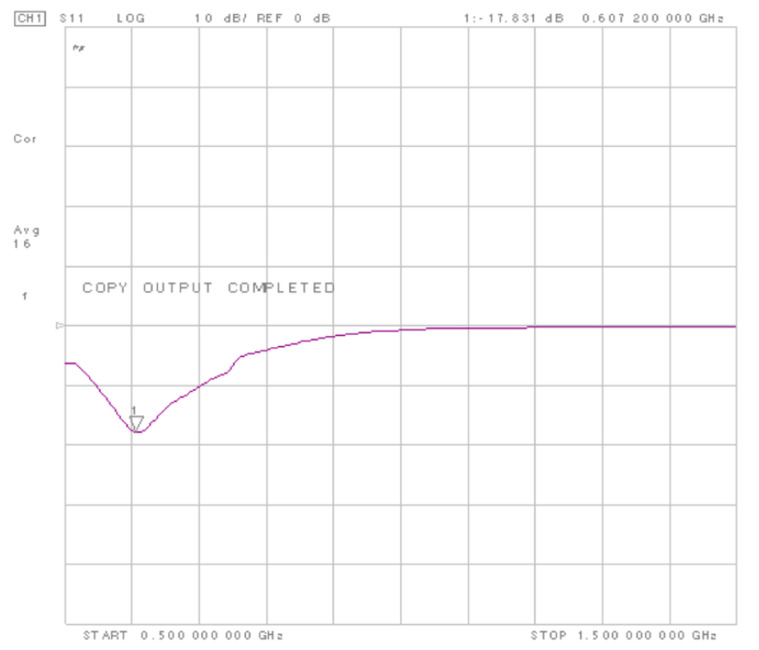
Measured input match (S11) of the RFED WuR. It can be seen that the input match is deviated to lower frequencies, with a valley centered at 607 MHz instead of 868 MHz.

The measured output of the WuR is 10 dB lower in magnitude in comparison with simulation results. This disagreement is caused by a deviation in the input matching network of the WuR. Figure 6 presents the measured input return loss of the circuit, obtained with an 8720ES network analyser. As seen, the input match has deviated to 607 MHz, causing the 10 dB losses observed at the WuR’s output. This shift can be originated by the tolerance of the discrete components and the variation of the inductance value with frequency, since the effective inductance value is expected to deviate to some extent at high frequencies.

### 2.3. WuR Validation

**Figure 7:**
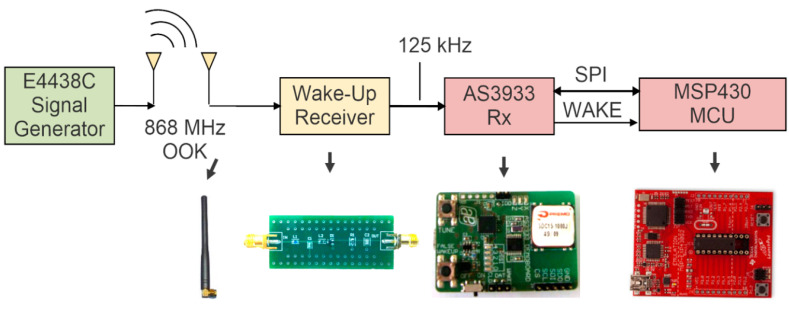
Scheme for the WuR prototype for validation.

The scheme used for WuR validation is the one represented in Figure 7. It is composed of the WuR and four main blocks: a pattern recognizer, a microcontroller, a transceiver and a T/R switch. The pattern recognizer is a low-power three-channel ASK receiver, formed by the AS3933 integrated circuit. The AS3933 is capable of generating an interrupt to activate the microcontroller unit and provides an integrated correlator capable of detecting a Manchester codification. The selected low-power MCU is the Texas Instruments MSP430G2553, with a mixed-signal 16-bit processor. The role of the transceiver is performed by a Texas Instrument CC1101 circuit, operating in the 868 MHz ISM band. The CC1101 is compatible with multiple modulation schemes, including OOK. The low-power ADG918 circuit is selected as T/R switch. A block diagram of the node prototype is shown in Figure 7. Since the main aim of the WuR is to generate the envelope of 125 kHz OOK signal for the AS3933, it is directly connected to one of its channels, and the functionality of the MSP430 allows programming the AS3933 via SPI. When the AS3933 identifies the correct 125 kHz wake-up signal, it generates a WAKE signal, which is sent to the microcontroller. The MCU receives this signal and an interrupt is generated to deactivate the low-power mode and begin the information exchange.

From the datasheets, the current drawn by the CC1101 and the ADG918 in sleep-mode are estimated to be 0.2 and 1 μA, respectively. On the other hand, in reception-mode, the CC1101 consumes 15 mA while the ADG918 maintains the same power consumption. If the power consumption of the development kits is de-embedded, the prototype is estimated to draw approximately 3 μA in sleep-mode (1.7 μA because of the AS3933, 0.1 μA due to the MSP430, 0.2 μA from the CC1101 and 1 μA coming from the ADG918) and 15.34 mA in reception-mode (1.7 μA because of the AS3933, 340 μA due to the MSP430, 15 mA from the CC1101 and 1 μA coming from the ADG918). The transceiver is operating at 1.2 kbauds in reception-mode with an input power above the sensitivity level of the CC1101. The system is capable of receiving a wake-up signal with a transmitting power of −5.5 dBm at a distance of 15 m. The proposed node utilizes a standard CR2032 battery with 230 mAh capacity at 3 V DC supply and 23 °C. The battery life depends mainly on the interval between wake-up signals and the time the node spends in reception-mode. It is possible to calculate the battery lifespan as shown in Equation (1), where *T_int_* is the interval between wake-up signals, *T_send_* is the time it takes to transmit a wake-up signal, *Q_bat_* is the battery capacity, *I_send_* represents the current consumption of the node while transmitting the wake-up signal and *I_sleep_* is the current drawn in sleep-mode. Taking into account the power consumption of the node and estimating an interval time of 100 s, the battery would last around one year. If the interval is increased to 900 s, the battery would last as long as five years.

(1) $$T_{dnode}=\frac{Q_{bat}\cdot T_{int}}{24\cdot \left(T_{send}\cdot I_{send}+I_{sleep}\cdot \left(T_{int}-T_{send}\right)\right)}$$

## 3. CMOS Integrated WuR Design

We show that a WuR with off-chip components can be developed to obtain a good performance. The design of WuRs with off-chip components can be advantageous since it is possible to implement high-Q input filters, improving the interference resilience of the receiver. However, the use of these bulky and costly resonators implies a higher bill of materials and assembly cost per device, which may be impractical in low-cost applications such as WSNs. Since integrability is one of the crucial aspects of WuR design, the development of a WuR with integrated CMOS technologies can be beneficial in terms of facilitating system integration and improving circuit performance. In this sense, a commercial UMC 65 nm CMOS process is selected for the development of an integrated RFED-based WuR. First, a MOSFET-based envelope detector is developed, and some modifications are added to improve the sensitivity and area of the WuR.

### 3.1. MOSFET-Based RFED WuR with Integrated Transformer

**Figure 8:**
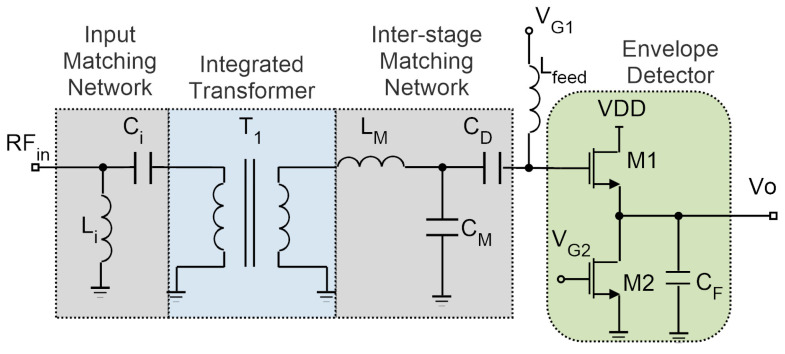
Schematic of the MOSFET-based RFED WuR with integrated transformer.

A MOSFET-based topology for the envelope detector is depicted in Figure 8. This structure is advantageous in terms of power consumption, but it achieves a limited sensitivity. 

**Figure 9:**
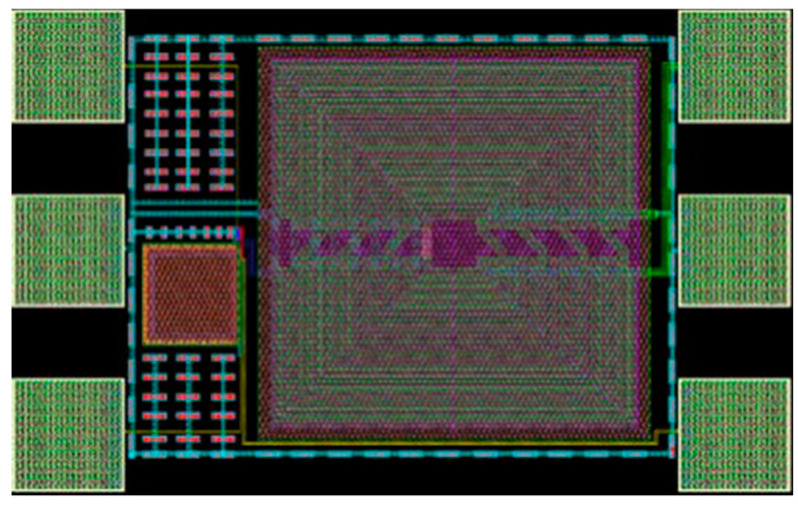
Layout of the MOSFET based RFED WuR with integrated transformer.

In this configuration, a common-drain amplifier is formed by transistor M1, biased in weak inversion so as to achieve a very low power consumption, and transistor M2 acts as a current source setting the drain current of M1. The values of V_G1_ and V_G2_ are adjusted to maintain very low power consumption with a reasonable gain. The drain current for the envelope detector is selected as 440 nA from a 1.2 V DC supply. The detector’s output impedance combined with capacitance C1 forms a low-pass filter, designed at a frequency of 125 kHz. Similar to [11,23,24], the principle of adding a large passive voltage gain before the envelope detector is exploited here to boost the WuR sensitivity. An integrated transformer can be included in the input matching network, as shown in Figure 8. This improvement comes at the expense of a higher circuit area due to the integrated inductors needed, but it introduces no additional power consumption. The transformer brings the advantage of additional voltage gain at the input, thus, increasing SNR before the envelope detection process. A transformer ratio of 1:8 is selected to provide a voltage gain of 18 dB, and an interstage matching network is needed to match the transformer’s output to the envelope detector’s high impedance input. The input matching network is formed by the series capacitor C_i_ and the shunt inductor L_i_, while the interstage matching network is composed of the shunt capacitor C_M_, and series inductor L_M_. The layout of the circuit is presented in Figure 9, with a total area of 634 × 391 μm^2^.

Table 1 shows a summary of device sizing and all components used. With this information, the circuit is simulated with the Advanced Design System software. An input return loss better than 40 dB is achieved at the frequency of 868 MHz. The full circuit is simulated with the matching networks and the envelope detector, achieving a sensitivity of −62 dBm.

### 3.2. Tuned RFED WuR with Integrated Active Inductor

**Figure 10:**
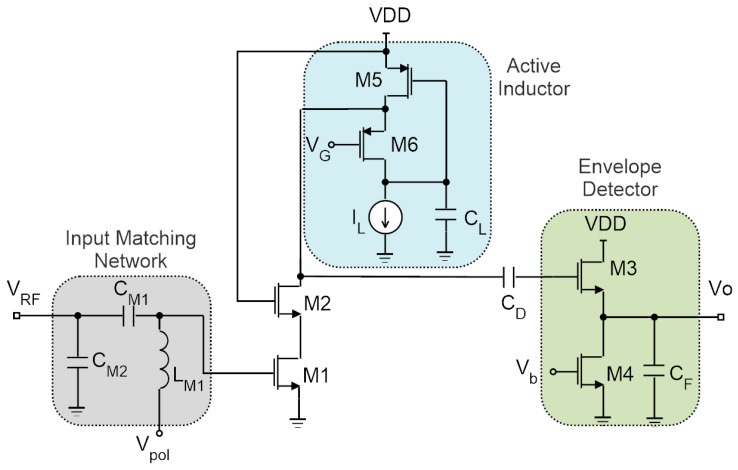
Schematic of the tuned RFED WuR with narrow-band cascode LNA and envelope detector, where the LC tank of the LNA is composed of an integrated active inductor.

The addition of a large passive gain effectively increases the sensitivity of the WuR without requiring additional power dissipation, but with a penalty in chip size. A possible solution to improve the WuR sensitivity with a slight increase in power consumption is to add an active gain stage prior to the envelope detector [29,30], and it is desirable to improve selectivity and noise figure by implementing a narrow-band amplifier. One possible topology to meet these requirements is shown in Figure 10. The conventional cascode LNA, composed of M1 and M2, can provide high gain while the LC tank implemented with an active inductor, formed by M5 and M6, provides narrow-band behavior with a low impact in circuit area.

The idea is to use an integrated PMOS version of a current-reuse active inductor composed of MOSFETs and capacitors as part of the LC tank to reduce the area penalty associated with integrated inductors [31]. The active inductor is composed of two PMOS transistors (M5 and M6), a capacitor *C_L_* and a current source that can be implemented by a single MOSFET. Assuming only *C_gs_* is being considered, the inductance, parallel capacitance, series resistance, parallel resistance and quality factor (*Q*) of the active inductor are given by the set of equations in (2). The small-signal input impedance of the circuit is given by Equation (3), where *r_o_*_5_ is the resistor modeling the channel length modulation of M5, *C_in_* is the capacitance seen at the circuit’s input, *g_ds_*_5_ and *g_ds_*_6_ represent the body-effect of M5 and M6, *C_L_* is equivalent to the parallel capacitance of the inductor, and *g_m_*_5_ and *g_m_*_6_ are the transconductances of transistors M5 and M6, respectively. The current source is left as an external pin for testing purposes. A gate voltage of 0.5 V is applied to bias transistor M5, and the current drawn by the active inductor is selected to be 10 nA so as to keep a very low power consumption. All the remaining components have the values seen in Table 2.

(2) $$L=\frac{C_{gs5}}{g_{m5}g_{m6}};C_{p}=C_{gs5};R_{s}=\frac{g_{ds5}+g_{ds6}}{g_{m5}g_{m6}};R_{p}=\frac{1}{g_{m6}};Q\approx \frac{R_{p}}{\omega L}$$

(3) $$Z_{i}\left(s\right)=\left(\frac{r_{o5}}{1+s\cdot r_{o5}C_{in}}\right)\|\left(\frac{g_{ds5}+g_{\mathrm{ds}6}}{g_{m5}g_{m6}}+s\frac{C_{L}}{g_{m5}g_{m6}}\right)$$

**Figure 11:**
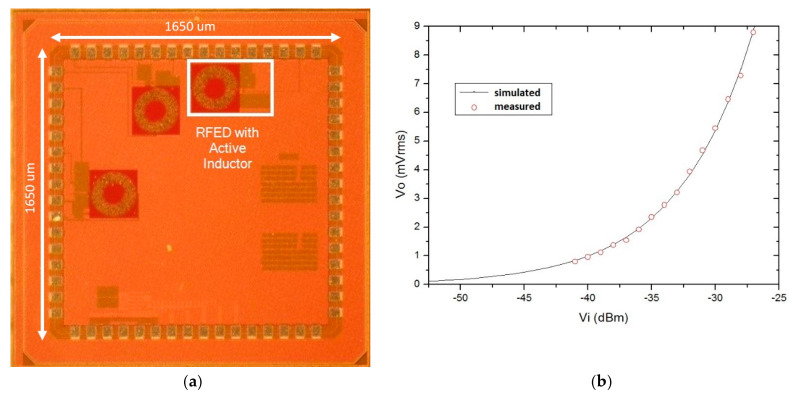
Layout (**a**) and measured sensitivity (**b**) of the RFED WuR with preamplifier and active inductor.

The effect of wire bonding and pad parasitic inductances and capacitances was taken into account for complete simulations of the circuit. The simulated input return loss has a value of 22.5 dB at 868 MHz, where the LNA has a maximum gain of 15 dB. The sensitivity of the full circuit is −57 dBm, with a power consumption of 40.08 μW. A microphotograph of the manufactured chip is presented in Figure 11a, with a total size of 272 × 464 μm^2^, excluding pads. As a consequence of inaccurate wire bonding models the input match of the circuit was deviated, and an external input matching network was necessary to perform the measurement setup of the circuit. A T-type matching network is added in series with the WuR’s input terminal, achieving an |S11| of 37 dB. With this, the sensitivity of the circuit is estimated to be around −55 dBm with a power consumption of 43.2 μW. The measurement results of the circuit sensitivity are shown in Figure 11b.

As predicted, in contrast with the previous solution the tuned-RF architecture achieves significant area savings thanks to the use of an integrated active inductor. This improvement comes at the expense of higher power consumption due to the added LNA, while keeping a similar sensitivity to the MOSFET-based RFED WuR with integrated transformer.

## 4. Overview and Discussion

**Figure 12:**
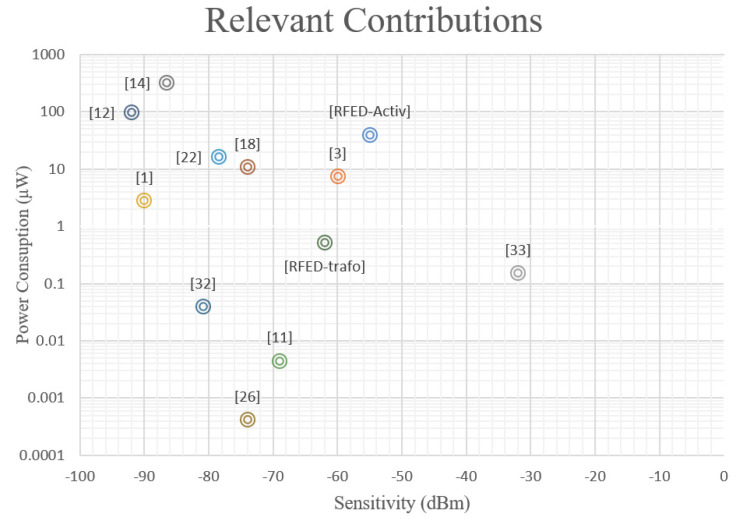
Representation of the performance of the proposed contributions along with some of the most relevant state-of-the-art works involving WuR design.

Three structures based on the radiofrequency envelope detection principle are explored. First, an off-chip RFED WuR is developed to demonstrate the feasibility of implementing a high-performance receiver with commercial off-the-shelf components. The advantage of this approach is that it can yield a higher selectivity compared to fully integrated solutions, mainly thanks to the possibility of implementing a high-Q input filter. However, the PCB manufacturing, assembly and related costs may not be suitable for low-cost applications such as WSNs. Therefore, the development of fully integrated WuRs in commercial CMOS technologies is considered. The first and simplest structure consists in a single MOSFET-based envelope detector with integrated transformer to improve the sensitivity without increasing power consumption. Thanks to the additional passive voltage gain, this solution achieves a sensitivity of −62 dBm with a power consumption of only 528 nW, but a penalty in circuit area is paid. The addition of a narrowband cascode LNA is also tested, but substituting the inductor in the LC tank by an integrated active inductor to save area. This results in a more compact solution, achieving a measured sensitivity of −55 dBm. In this case, a sacrifice in power consumption is present and the circuit obtains a similar performance to the previous solution with a power dissipation of 40 μW. A comparison between our contributions and some of the most relevant state-of-the-art solutions is presented in Figure 12, where the x- and y-axis represent sensitivity and power consumption, respectively. The label RFED-trafo in the figure indicates the RFED WuR with integrated transformer, and the label RFED-Activ represents the RFED with integrated active inductor.

A detailed comparison of circuit performance is given in Table 3. The first definition of the figure of merit (FoM_LAT_), extracted from [11], is calculated as shown in Equation (4), where *P_SEN_* corresponds to the sensitivity in dBm and *P_DC_* is the power consumption in dBm. This computation of the FoM is particularly suitable taking into account that the two most relevant parameters in low-average throughput WSN applications are power consumption and sensitivity [11]. In applications where the throughputs of the node and data rate are important performance metrics, a modified version of the FoM that accounts for the data rate can be used. Note that in Equation (4) other parameters such as the operating frequency, area and cost per node are not considered to be relevant. The second definition of the figure of merit (FoM_ARE_), presented in Equation (5), accounts for the area contribution of each circuit, so that a higher area affects negatively to the FoM.

(4) $${\mathrm{FoM}}_{\mathrm{LAT}}\left(\mathrm{dB}\right)=-P_{SEN}\left(\mathrm{dBm}\right)-P_{DC}\left(\mathrm{dBm}\right)-60$$

(5) $${\mathrm{FoM}}_{\mathrm{ARE}}\left(\mathrm{dB}\right)=-P_{SEN}\left(\mathrm{dBm}\right)-P_{DC}\left(\mathrm{dBm}\right)-10*\log\left(area\left({\mu m}^{2}\right)\right)$$

It can be seen in Table 3 and in Figure 12 that the RFED with integrated transformer is superior to the proposed RFED with off-chip components and the integrated RFED with active inductor, as revealed by the two FoMs used. It can be seen that the RFED with integrated transformer is superior to most of the other solutions, only [12,22,26,32] show a higher FoM_ARE_. The RFED with integrated transformer shows a lower value of the FoM_ARE_ because we integrated the input matching network and its area is included as part of the circuit. In contrast, the solutions proposed by [22,26,32] only account for the area of the integrated WuR, although they employ high-Q external input matching networks. In [12], two external inductors are used as well for input match and clock generation, but their area is not included. To compare the circuits in similar conditions, the area of the input matching network is removed from the RFED WuR with active inductor, resulting in a total footprint of 165 × 107 μm^2^, the lowest area reported, to our knowledge.

The solution proposed by [3] is an RFED WuR implemented with off-chip components and a structure similar to that proposed in Section 3. The authors use an envelope detector with Schottky diodes and the AS3933 circuit as pattern recognizer as well. In [33] the authors propose an RFED WuR, too. The WuR is implemented in a PCB along with the TI CC430F5147 SoC, containing an MCU and a transceiver, and it has two different antennas to improve measurement accuracy instead of sharing the path with a T/R switch, as we did in Section 2. A tuned-RF topology is selected in [1] to implement a WuR with excellent performance, where a modified MAC protocol and enhanced duty-cycled listening are employed to reduce power consumption while keeping a reasonable latency. This work presents a prototype of the proposed WuR to validate the results where all the blocks are assembled together in a single PCB. The authors in [22] present a fully integrated subsampling-based WuR with a high sensitivity and a slightly higher power consumption. However, the proposed RFED WuR transformer achieves a high sensitivity with less than ten-fold power consumption. An RFED WuR with integrated transformer is presented in [11], achieving a higher sensitivity than the circuit proposed in Section 3.1 with a power consumption of only 4.5 nW, mainly due to the implementation of a higher passive gain of 25 dB and a meticulous design in terms of power consumption. An uncertain-IF WuR is demonstrated in [12]. This WuR presents a power consumption as high as 99 μW that may be unsuitable for WSN applications, but still the sensitivity is as high as −97 dBm, which is by far the best sensitivity that has been reported, to the best of the authors’ knowledge. An injection-lock architecture is adopted in [18], where the authors implement an external loop antenna, thus being able to present two operation modes with low power consumption and high sensitivity. The SRO topology is used in [14], resulting in a very large power dissipation with a very high sensitivity. Another fully integrated WuR is shown in [26], where the receiver employs gate-biased self-mixers and an efficient strategy to implement matched filters with DC offset cancellation. The WuR is composed of a 40-stage self-mixer to achieve a very high sensitivity with a power consumption below 1 nW. Finally, in [32] the authors propose a code domain matched filtering technique by using a continuous-time analog correlator, which results in a WuR with a very high performance. 

We can conclude that the reported circuits show a performance in line with most of the state-of-the-art contributions with a very low area.

## 5. Conclusions

In this paper, we present the development of three different WuRs for densely populated WSNs based on the envelope detection architecture, all of them operating at the 868 MHz ISM band. The aim of this work is to provide high integrability with a low cost-per-node, so as to facilitate the implementation of sensor nodes in low-cost IoT applications. In order to demonstrate the feasibility of implementing a WuR with commercially available off-chip components, the design of an RFED WuR in a PCB mount is presented. The circuit is validated in a real scenario by testing the WuR in a system with a pattern recognizer (AS3933), an MCU (MSP430G2553 from TI), a transceiver (CC1101 from TI) and a T/R switch (ADG918). The WuR has no active components and features a sensitivity of about −50 dBm, with a total size of 22.5 × 51.8 mm^2^ for the resulting PCB. However, the nature of this WuR may be incompatible with small nodes and low-cost applications because of the low level of integrability and assembly costs. Therefore, to facilitate the integration of the WuR in a real system, a design in a commercial UMC 65 nm CMOS process is explored. Firstly, an RFED with an integrated transformer providing a passive voltage gain of 18 dB is demonstrated. The circuit achieves a sensitivity as low as −62 dBm from simulations and a power consumption of only 528 nW, with a total area of 634 × 391 μm^2^. Secondly, to reduce the area of the circuit a design of a tuned-RF WuR with an integrated active inductor is presented. In this case, the WuR features a sensitivity of −55 dBm with a power consumption of 43.5 μW and a total area of 272 × 464 μm^2^, obtaining a significant area reduction at the cost of a higher power consumption when compared to the WuR with integrated transformer. However, the area of this circuit is given with an integrated input matching network, which is frequently implemented externally. If we account for the circuit’s core by removing the area of the input matching network, the RFED with active inductor presents an area of only 165 × 107 μm^2^, the lowest area reported, to our knowledge. The other alternatives discussed show a performance in line with most of the state-of-the-art contributions with a very low die footprint, making the topologies attractive in situations where a high integrability and low cost-per-node is pursued.

## Figures and Tables

**Table 1:** Summary of component values for the MOSFET-based envelope detector with transformer.

Parameter	Value	Parameter	Value
M1	(25 μm/0.3 μm)	C_M_	4.2 pF
M2	(10 μm/3 μm)	L_i_	75 nH
C_D_	10 pF	L_M_	10 nH
C_F_	10 pF	V_G2_	0.5 V
C_i_	1.41 pF	V_DD_	1.2 V

**Table 2:** Summary of component values for the tuned RFED WuR with active inductor.

Parameter	Value	Parameter	Value
M1, M2	2 × (1 μm/0.12 μm)	C_M1_	11 pF
M3	(25 μm/0.3 μm)	C_M2_	3.67 pF
M4	(10 μm/3 μm)	L_M1_	10 nH
M5	(0.5 μm/0.18 μm)	V_b_	0.56 V
M6	(0.6 μm/0.18 μm)	V_G_	0.5 V
C_F_	10 pF	V_pol_	0.76 V
C_D_	10 pF	V_DD_	1.2 V
C_L_	9.81 pF		

**Table 3:** Performance summary of the explored RFED-based solutions.

WuR Topology	Frequency (GHz)	Sensitivity (dBm)	Power Consumption (μW)	Area (μm^2^)	FoM_LAT_ (dB)	FoM_ARE_ (dB)
PCB RFED	0.868	−50	-	22,500 × 51,800	N/A	N/A
RFED w/transformer	0.868	−62	0.528	634 × 391	94.7	40.8
RFED w/active inductor	0.868	−55	43.2	272 × 464	68.9	17.9
[3] RFED	0.868	−60	7.5	37,000 × 22,000	81.2	−7.8
[33] RFED	0.868	−32/−55	0.152/1.2	--	70.2/84.2	N/A
[1] Tuned-RF	0.868	−90	2.8	46,300 × 24,500	115.5	25
* [22] Sub-sampling	0.915	−78.5	16.4	1000 × 200	96.4	43
[11] RFED w/transformer	0.1135	−69	0.0045	(PCB)	122.5	N/A
* [12] Uncertain-IF	2.4	−97/−92	99	360 × 160	107.0/102.0	54.4
[18] Injection-Lock	0.433	−80/−74	54/11	900 × 500	91.4/93.6	37
[14] SRO	0.915	−86.5	320	900 × 500	91.4	34.9
* [26] Self-mixer + DLL	0.151/0.434/1.016	−79/−79.2/−74	~0.00042	370 × 250	142.8/143/137.8	88.1
[32] CT-Analog Correlator	0.4508	−80.9	0.04	500 × 480	124.9	71.1

* Input matching network implemented externally.
